# Phase change composite based on protic ionic liquids 2-hydroxyethylammonium lactate and stearic acid for thermal energy storage systems at intermediate temperatures

**DOI:** 10.1038/s41598-025-08514-0

**Published:** 2025-07-07

**Authors:** Masumeh Mokhtarpour, Hemayat Shekaari, Ali Rostami, Saeid Faraji

**Affiliations:** 1 Department of Physical Chemistry, University of Tabriz, Tabriz, Iran; 2 Photonics and Nanocrystal Research Lab (PNRL), Faculty of Electrical and Computer Engineering, University of Tabriz, Tabriz, Iran; 3 School of Engineering and Materials Science, Queen Mary University of London, London, UK

**Keywords:** Thermal energy storage, Ionic liquids, Fatty acid, Phase change materials

## Abstract

The incorporation of phase change materials (PCMs) within thermal energy storage (TES) systems represents a pivotal advancement in materials science, enabling the efficient harnessing and deployment of solar energy and waste heat. PCMs based on ionic liquids hold significant importance in various fields and applications due to their unique properties and advantages. The new family of phase change composites based on ionic liquids, (ILs) tris(2-hydroxyethylammonium) lactate ([THEA]La), bis(2-hydroxyethylammonium) lactate ([BHEA]La), and 2-hydroxyethylammonium lactate ([HEA]La) with stearic acid (SA), are presented in this work. These PCMs offer a secure and cost-effective capacity and function between 323 and 423 K. The flexibility to control chemical and phase characteristics, combined with good chemical and thermal stability, give ILs an inherent “green” quality that makes them ideal for PCMs. Using PCM1 ([HEA]La/Sa) the effective latent heat 245 J g^−1^. The PCM1 ([HEA]La/SA) was obtained according to the results of differential scanning calorimetry method which has a greater heat capacity rather than the other two PCMs. Thermal stability analysis indicates that PCM1 ([HEA]La/SA) has the highest level of stability (97.21%). This study delves deeper into the molecular underpinnings responsible for the impressive thermal energy storage capabilities exhibited by these ionic materials, elucidating their structural intricacies and highlighting the pivotal role of hydrogen bonds in enhancing PCM performance. By adopting this approach, we sidestep the intricacies and expenses associated with composite PCMs, relying instead on readily available and cost-effective materials.

## Introduction

The continued reliance on fossil fuels as a primary energy source has been identified as a contributing factor to several critical global issues, such as climate change^1^, ecosystem degradation^2^, and public health risks from poor air quality^3^. The effective utilization of energy is a pressing concern due to anxieties surrounding greenhouse gas emissions and the depletion of fossil fuel reserves. Furthermore, the inherent unsustainability of fossil fuels, stemming from their rapidly diminishing supplies, underscores the urgency and necessity of transitioning to a carbon-free energy sector^4^.

It is advised to create new renewable energies and boost energy efficiency as the global energy supply and demand gap widens. The issue of effective energy utilization may be addressed through the implementation of latent heat storage, a form of thermal energy storage (TES) technology. This approach can help mitigate the variability of energy demand across time and location^5,6^. Due to high potential of phase change materials (PCMs) for temperature regulation and heat storage, PCM play an important role in various application fields such as thermal energy storage, solar energy, technical textiles, smart materials, non-volatile memories and greenhouses^7,8^.

The energy crisis of 1973–1974 served as a pivotal turning point, whereby energy management strategies became increasingly reliant on PCMs for TES. Across various industrial and domestic sectors, the utilization of PCMs as a TES solution has emerged as an elegant and practical approach to enhance the efficiency of thermal energy storage and recovery. By integrating PCM-based TES systems, the capture and subsequent deployment of waste thermal energy can be optimized, thereby improving the overall energy efficiency and sustainability within these sectors^9–12^.

Ionic liquids (ILs), or low-melting salts made up of cations and anions, are being studied. With wide electrochemical potential window, chemical durability, high ionic conductivity, low toxicity, high thermal stability, and negligible vapor pressure, ILs have been known as compatibilizer^13^, flame retardant^14^ and green solvent in wide areas^15^.

Considering that certain ILs demonstrate low flammability, corrosivity, and volatility—attributes typically regarded as drawbacks of conventional PCMs^16^, it is becoming increasingly apparent that these characteristics confer advantages to PCMs. For TES applications, many different ILs have been used as PCMs^17–21^. Additionally used as thermal energy storage devices, hydroxyethyl ammonium based ILs are of crucial relevance^22,23^.

Due to their potential cheap cost, the acid–base family of protic ILs produced via a straightforward acid base reaction is of considerable interest. Among protic ILs, PCMs based on ammonium^24^, pyrazolium^21^ and guanidinium^25,26^ cations have been studied to this point. These materials have high energy storage capabilities, and have melting points in the intermediate, desired temperature range.

As a result, ILs have yet to fully realize their promise as PCMs, and there is a sizable research knowledge gap on the links between their structure and properties. Protic ionic liquids can also occasionally exhibit extra solid–solid phase transitions. While they play a significant role in the creation of new PCMs, the molecular causes of the occurrence of solid–solid phase transitions and their impact on melting enthalpy remain poorly understood^27^.

Distributed thermal energy storage (TES), on the other hand, is unexpectedly underdeveloped and ignored in this situation. Thermal energy applications, such as heating or cooling, account for between 30 and 50% of total energy consumption worldwide, offering a situation with significant potential for TES technology^28,29^. In terms of cost, reliability, and durability, it is increasingly evident that TES can rival electrical and chemical energy storage systems for both home and commercial energy storage^30^.

In contrast, latent heat storage systems use reversible phase transitions to store and release thermal energy, most frequently the solid–liquid (melting and crystallization) transition. PCMs may be the TES technology that is most suited for extensive renewable energy storage^30^.

There is no longer a need for fossil fuels because fatty acids may be obtained from living things^31^. They are non-toxic, affordable, and resistant to corrosion, among many other positive attributes. In addition, they frequently have high latent heat, favorable phase transition temperatures, superior chemical and thermal stability, and no phase separation or supercooling^32–34^. Fatty acid micro-crystallites play a crucial role in the ability of PCMs to function as heat storage blankets, effectively regulating the temperature within an enclosure. Fatty acids, with high latent heat of fusion, prove to be valuable PCMs. The addition of stearic acid allows for the incorporation of various functionalities into composite PCMs^35,36^.

Solar power plants represent a key category among renewable energy sources for high-capacity electricity generation on a national scale, according to the international community. However, solar panels face the challenge of diminished or even disrupted performance in the absence of sunlight. To address this issue, effective PCMs have been employed. Through innovative design, PCMs will be leveraged to assess and enhance the thermal performance of solar panels, ensuring continued electrical compensation even in conditions with limited sunlight^37–40^.

In continuation of our previous work related to ILs^41–46^, the 2-hydroxyethylammonium-based ILs have been chosen as special ILs in this research because their heat capacity is larger than that of other ILs. In this paper, we examine the most recent PCMs for TES applications in power-generating solar cells, along with ongoing initiatives to create novel PCMs with improved security and performance. PCMs based on ILs tris(2-hydroxyethylammonium) lactate, bis(2-hydroxyethylammonium) lactate, 2-hydroxyethylammonium lactate and stearic acid were developed as thermal energy storage materials. X-ray diffraction (XRD), scanning electron microscopy (SEM), and fourier transformation infrared spectroscopy (FT-IR) were used to analyze the microstructure and chemical composition of PCMs. The latent heat, specific heat capacity, and thermal stability of the PCMs were determined using differential scanning calorimetery (DSC) and thermogravimetric analysis (TGA) studies.

## Experimental measurements

### Chemicals

The suppliers of lactic acid, ethanolamines, and stearic acid were Shazand Petrochemical Co., Sigma Aldrich, and Merck Co. Lactic acid was used to neutralize monoethanolamine, diethanolamine, and triethanolamine in order to create tris(2-hydroxyethylammonium) lactate, bis(2-hydroxyethylammonium) lactate, and 2-hydroxyethylammonium lactate ILs. These ILs underwent no further filtration. In order to perform the synthesis, ethanolamines were added to a glass flask with three necks and a reflux condenser. Lactic acid was then introduced dropwise, and the mixture was stirred with a magnetic stirrer for 24 h at room temperature to complete the reaction. Detailed information on all compounds can be found in Table 1.

**Table 1:** Information of used chemicals.

Chemicals	Source	CAS No	Molar mass (g∙mol^-1^)	Mass percent (purity)
Monoethanolamine [MEA]	Shazand petrochem	141-43-5	61.08	˃ 99
Diethanolamine [DEA]	Shazand petrochem	111-42-2	105.14	≥ 98.5
Triethanolamine [TEA]	Shazand petrochem	102-71-6	149.19	˃ 99
L-(+)-lactic acid (La)	Sigma Aldrich	79-33-4	90.08	≥ 98
2-hydroxyethylammonium lactate [HEA]La	Synthesized in our lab	68,81569-0	151.16	˃ 99
Bis(2-hydroxyethyl)ammonium lactate [BHEA]La	Synthesized in our lab	–	195.22	˃ 98
Tris(2-hydroxyethyl)ammonium lactate [THEA]La	Synthesized in our lab	20475-12-1	239.27	˃ 97
Stearic acid (SA)	Merck	57-11-4	284.48	≥ 99

The suppliers were provided the purities of the used components.

### Preparation of phase change composites (PCMs) based on ionic liquids and stearic acod

The ionic liquids synthesized in the previous section were mixed with stearic acid (1:1 mol ratio) to prepare PCMs. Ionic liquids and stearic acid can simultaneously act as hydrogen bond acceptors and donors in a tightly sealed glass vial at 373 K until a uniform clear solution is formed.

### Characterization of phase change composites (PCMs)

The FT-IR transmittance spectra for the prepared PCMs were acquired using Bruker’s Tensor 270-KBr. Also, an XRD instrument (Tongda, TD 3700, Cu Kα, λ = 1.54 Å) was employed to analyze the X-ray diffraction patterns of the PCMs. The microstructure and morphology of the prepared PCMs were examined using a scanning electron microscope (TESCAN, MIRA3 FEG-SEM).

### Thermal analyses of phase change composites (PCMs)

Calorimetric measurements were conducted to determine the thermophysical properties of the prepared PCMs, including PCM3 (tris(2-hydroxyethylammonium) lactate/stearic acid), PCM2 (bis(2-hydroxyethylammonium) lactate/stearic acid), and PCM1 (2-hydroxyethylammonium lactate/stearic acid). A DSC (Netzsch DSC-200 F3) was used for thermal studies in order to determine the PCMs’ specific heat capacity and heat of fusion up to 373 K. The PCMs were heated progressively to 373 K in order to accomplish this. The thermal characteristics of the PCMs, such as their heat of fusion and specific heat capacity, could be more easily determined thanks to this heating procedure. With a heating rate of 10 K/min, specific heat capacity tests were done between 298.15 K and 373.15 K. An electronic balance was used to measure the weights of PCM samples with a 10^−3^ g accuracy. The sensitivity of the used DSC equipment is 10^−5^ mV/W^−1^.

Using a TGA device (METTLER TOLEDO, TGASDTA 851e), the thermal stability of the prepared PCMs was evaluated. The analysis was carried out between 50 and 700 °C in a nitrogen environment (N_2_, 30 mL/min^−1^).

## Results and discussion

Due to their extremely stable chemical and thermal characteristics, fatty acids and ionic liquids (ILs) make good PCMs^26^. Following up on our earlier research^47,48^, advanced PCMs based on a combination of stearic acid and 2-hydroxyethylammonium-based ILs were investigated.

### Characterization results

**Fig. 1:**
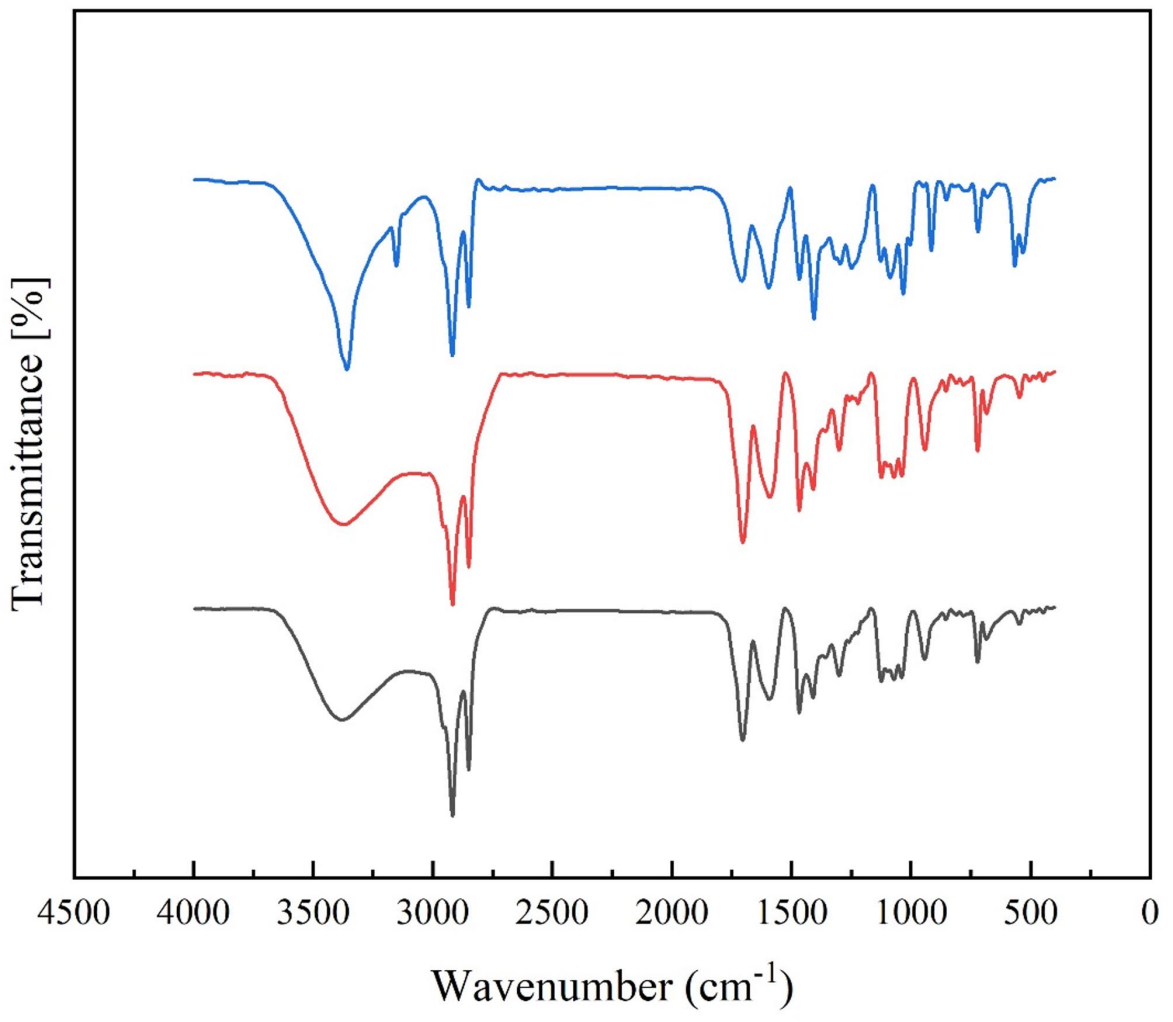
The FT-IR spectra of the; (black) PCM1 ([HEA]La/SA), (red) PCM2 ([BHEA]La/SA), (blue) PCM3 ([THEA]La/SA).

Figure 1 displays the FT-IR spectra of the PCMs, specifically PCM3 (tris(2-hydroxyethylammonium) lactate/stearic acid), PCM2 (bis(2-hydroxyethylammonium) lactate/stearic acid), and PCM1 (2-hydroxyethylammonium lactate/stearic acid). The characteristic FT-IR peaks for the prepared PCMs are detailed as follows:

FT-IR (KBr, cm^−1^) for PCM1 ([HEA]La/SA): 3367, 2917, 2849, 1704, 1591, 1467, 1410, 1300, 1125, 1072, 1039, 943, 721, and 543.

FT-IR (KBr, cm^−1^) for PCM2 ([BHEA]La/SA): 3376, 2917, 2849, 1704, 1590, 1467, 1410, 1300, 1124, 1071, 1038, 942, 721, and 547.

FT-IR (KBr, cm^−1^) for PCM3 ([THEA]La/SA): 3357, 3152, 2918, 2849, 1708, 1595, 1466, 1406, 1295, 1127, 1089, 1032, 948, 720, and 566.

As depicted in Fig. 1, the PCMs exhibit identical peaks to those of the ionic liquids and stearic acid, given their shared molecular structure. These shared characteristics are partly attributed to stearic acid, which is a highly saturated fatty acid. Within the range of 543 to 721 cm^−1^, the absorption peak of COO^−^ is a significant material property indicator. Moreover, long-chain alkyl groups are indicated by the strong and characteristic peaks between 2849 and 2917 cm^-1^, which correspond to the symmetric and antisymmetric vibrational modes of CH_2_, respectively. Notably, the C = O peak of carboxylic acid carbonyls falls within the range of 1704 to 1709 cm^-1^. Additionally, two relatively weak peaks are observed within the range of 1072 to 1089 cm^-1^, indicating the antisymmetric stretching vibration peaks of CH_3_ groups.

The vibrational behavior of the carboxyl (–COOH) functional group in stearic acid (SA) can be analyzed using infrared spectroscopy. The characteristic peaks observed in the spectrum correspond to different types of bending vibrations of the carboxyl group:

- **In-plane bending vibration (~ 948 cm**^**−1**^**)**: This mode occurs when the carboxyl group undergoes deformation within the plane of the molecule. In other words, the –COOH group bends while remaining in the same geometric plane as the surrounding molecular structure. This type of vibration typically arises due to interactions between the carboxyl group and neighboring molecular components.
- **Symmetric bending vibration (~ 1467 cm**^**−1**^**)**: In this mode, both oxygen atoms of the carboxyl group move synchronously in a way that maintains overall symmetry. This vibration is commonly associated with the stretching and contraction of the C = O and C-O bonds in a balanced manner, which is often influenced by hydrogen bonding and molecular packing.
- **Out-of-plane bending vibration (~ 1300 cm**^**−1**^**)**: This type of vibration involves the movement of the carboxyl group perpendicular to the molecular plane. Unlike in-plane bending, where the motion is constrained to a single plane, out-of-plane bending involves a twisting or wagging motion of the –COOH group, often affected by intermolecular forces and the surrounding molecular environment.

These vibrations are crucial for identifying the presence of carboxyl groups in stearic acid and understanding their molecular interactions in different environments, such as self-assembled monolayers or complex lipid structures^49^. Meanwhile, N–H, which is connected to ionic liquids, has a peak in the region of 3357 to 3376 cm^-1^. These results offer strong experimental support for the idea that blending ionic liquids with SA does not result in any chemical reactions. They also show that the final PCMs have the same chemical characteristics as the original materials^50^.

**Fig. 2:**
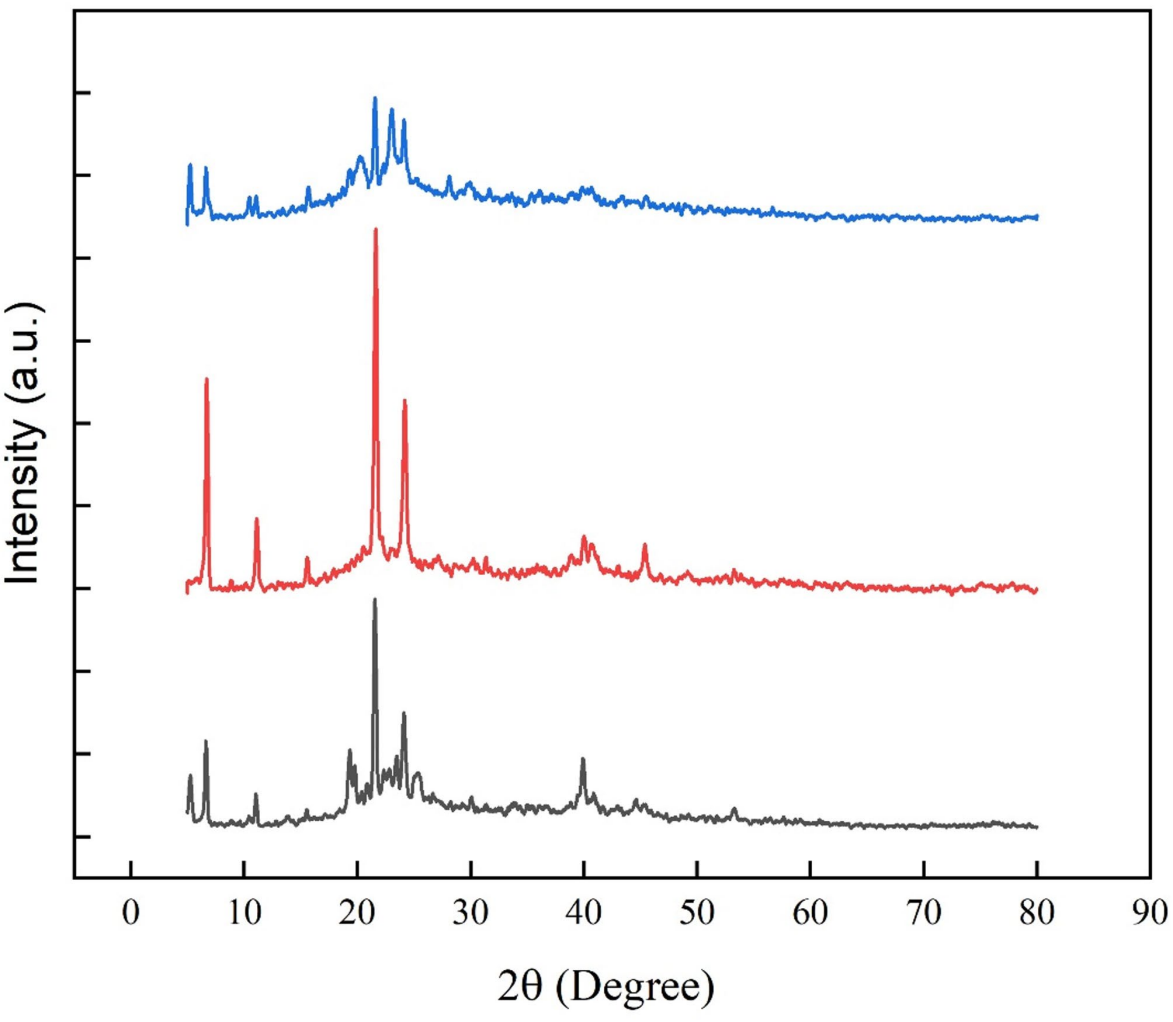
The XRD patterns of the; (black) PCM1 ([HEA]La/SA), (red) PCM2 ([BHEA]La/SA), (blue) PCM3 ([THEA]La/SA).

The XRD patterns of PCM1 ([HEA]La/SA), PCM2 ([BHEA]La/SA) and PCM3 ([THEA]La/SA) are shown in Fig. 2. The scanning rate and the scanning angle are 5°/min and 5 − 80°, respectively. The angles of the indicator peaks for PCM1 ([HEA]La/SA) are 7.12, 11.32, 16.14, 19.48, 21.61, 23.72, 25.77, 40.41°. The angles of the obvious peaks of PCM2 ([BHEA]La/SA) are 7.82, 11.46, 16.71, 19.69, 21.92, 23.80, 25.94, 41.23° and the angles associated with the characteristic peaks of PCM3 ([THEA]La/SA) are as follows: 8.11°, 11.61°, 16.84°, 19.94°, 21.86°, 23.87°, 26.21°, and 41.34°. These angles correspond to the fundamental phases attributed to fatty acids and remain consistently stable. These observations indicate that the PCMs maintain a stable combination and homogeneity without the presence of any new phases.

Polymorphs, or various crystal forms, are adopted by fatty acid molecules^51^. The geometry of these configurations differs from the symmetry of the unit cell. While some of these polymorphs can be metastable, others can be stable at room temperature^35^. The processing conditions have a significant impact on the detected polymorphs. Fatty acid crystallinity has drawn a lot of interest^42^. At 2θ values of around 10° or less, the primary XRD reflections from fatty acids may be detected and some higher-order thoughts appear at higher 2θ values.

Due to its hydroxyethyl branch, PCM3 ([THEA]La/SA) offers a larger variety of polymorphism options than PCM1 ([HEA]La/SA) and PCM2 ([BHEA]La/SA). Fatty acid crystallinity has drawn a lot of interest^52^.

**Fig. 3:**
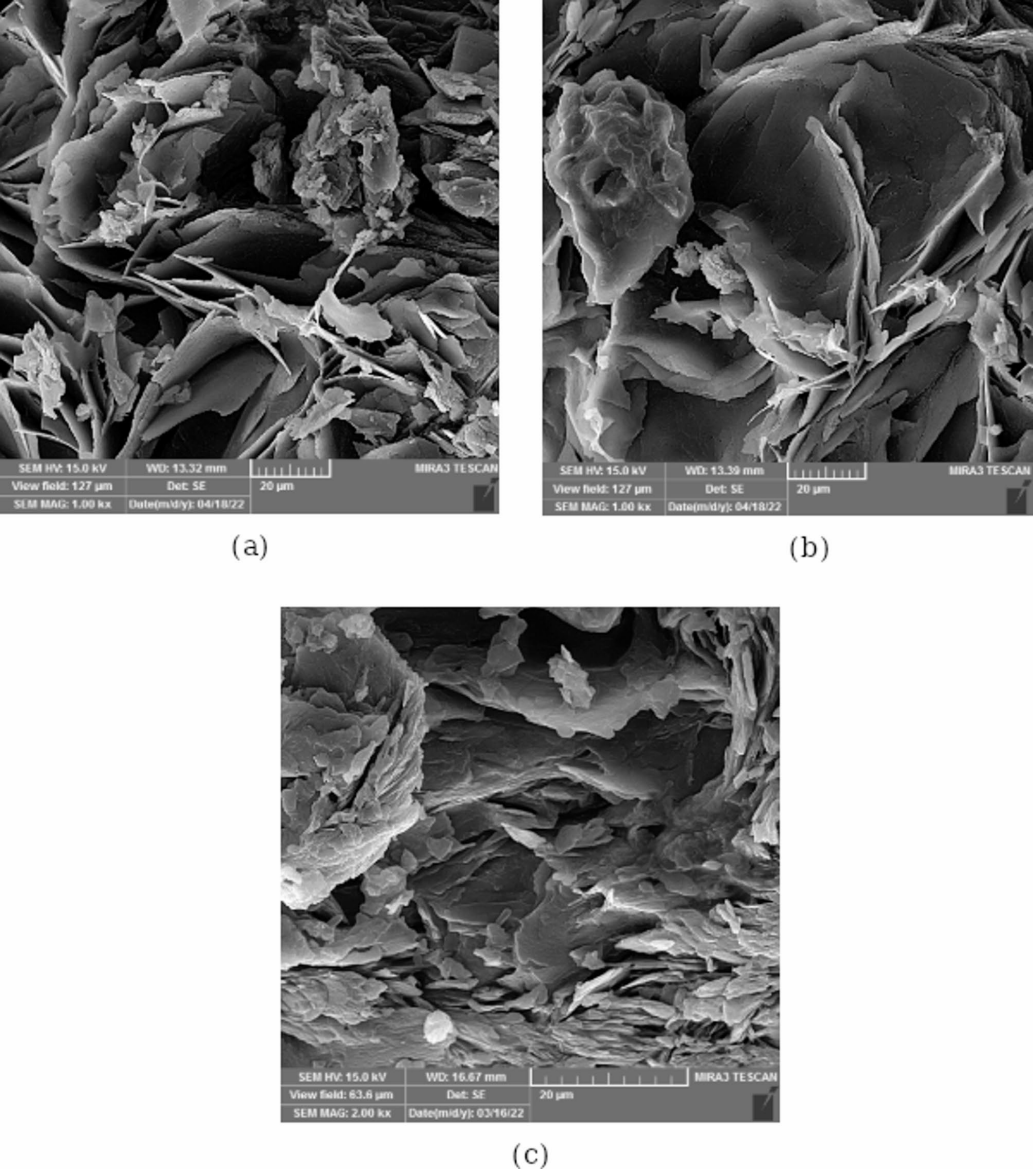
The SEM images of the; (**a**) PCM1 ([HEA]La/SA), (**b**) PCM2 ([BHEA]La/SA), (**c**) PCM3 ([THEA]La/SA).

Figure 3 shows the SEM photos of PCM1 ([HEA]La/SA), PCM2 ([BHEA]La/SA) and PCM3 ([THEA]La/SA). PCMs have a layered structure and a rough surface, as seen in the Fig. 3^31^. It can be seen that stearic acid is well mixed with ILs. The surface tension forces of stearic acid combine with the ionic force in the network structure of ILs to increase the mechanical strength of PCMs. Additionally, the PCM1 ([HEA]La/SA) has smaller size and more homogeneous microstructures than PCM2 ([BHEA]La/SA) and PCM3 ([THEA]La/SA). The composites created utilizing ILs and fatty acids are all layered and in the shape of sheets, as can be seen from the SEM pictures, and have a larger surface ratio than the basic ingredients that were used to melt and freeze them.

### Thermal results

**Fig. 4:**
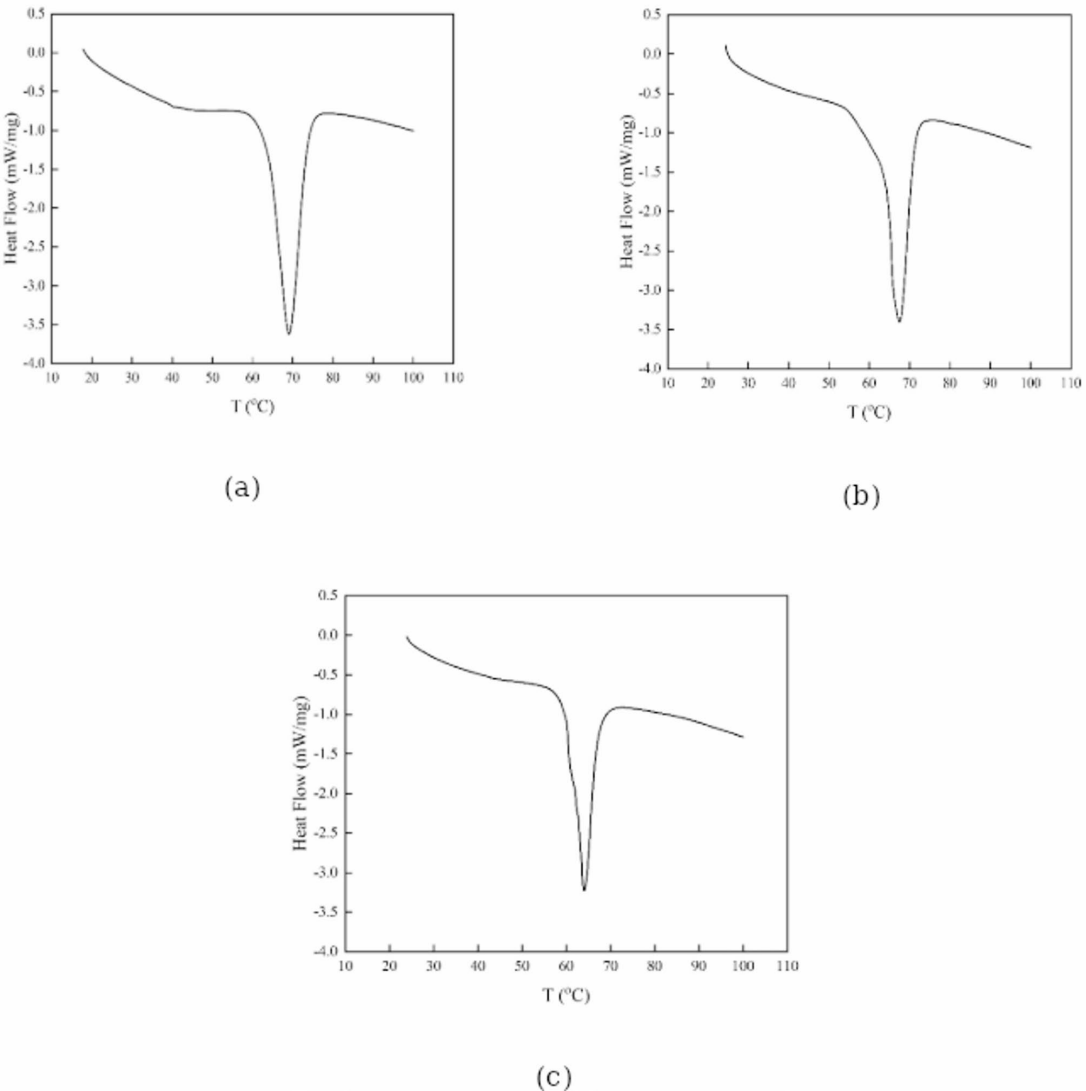
The DSC analysis of the; (**a**) PCM1 ([HEA]La/SA), (**b**) PCM2 ([BHEA]La/SA), (**c**) PCM3 ([THEA]La/SA).

Calorimetric results for PCMs are illustrated in Fig. 4 and Table 2. The melting points of PCM1 ([HEA]La/SA), PCM2 ([BHEA]La/SA), and PCM3 ([THEA]La/SA) were recorded at 342.21, 340.64, and 337.13 K, respectively. The results show that PCM1 ([HEA]La/SA), which melts with more energy than the others, has a stronger interactions.

**Table 2:** DSC and TGA data of the stearic acid, PCM1 ([HEA]La/SA), PCM2 ([BHEA]La/SA) and PCM3 ([THEA]La/SA).

Chemicals	Melting point (K)	Latent heat (kJ·kg^-1^)	Thermal stability or residue amount (%) up to 423.15 K
Stearic Acid	342.55^50^ 342.35^51^	199^50^ 203^51^	–
PCM1 ([HEA]La/SA)	342.21	245	97.17
PCM2 ([BHEA]La/SA)	340.64	231	96.80
PCM3 ([THEA]La/SA)	337.13	157	95.66

Thermal conductivity of ILs is [HEA]La = 0.255, [BHEA]La = 0.236 and [THEA]La = 0.226 (W⋅m^−1^ K^−1^) ^47^.

The results of this table show stearic acid and PCM phase transition properties are quite similar. Furthermore, Table 2 demonstrates that the latent heat values for PCM1, PCM2 and PCM3 are 245 kJ·kg^-1^, 231 kJ·kg^-1^ and 157 kJ·kg^-1^, respectively. The latent heatsof PCMs are increased by the stronger hydrogen bond formation between the NH_2_ group of the PCM and the COOH group of stearic acid. From a physical perspective, the formation of hydrogen bonds in PCMs can be attributed to the presence of hydroxyl and carboxyl functional groups. These groups create dipole–dipole interactions, which facilitate hydrogen bonding.

**Fig. 5:**
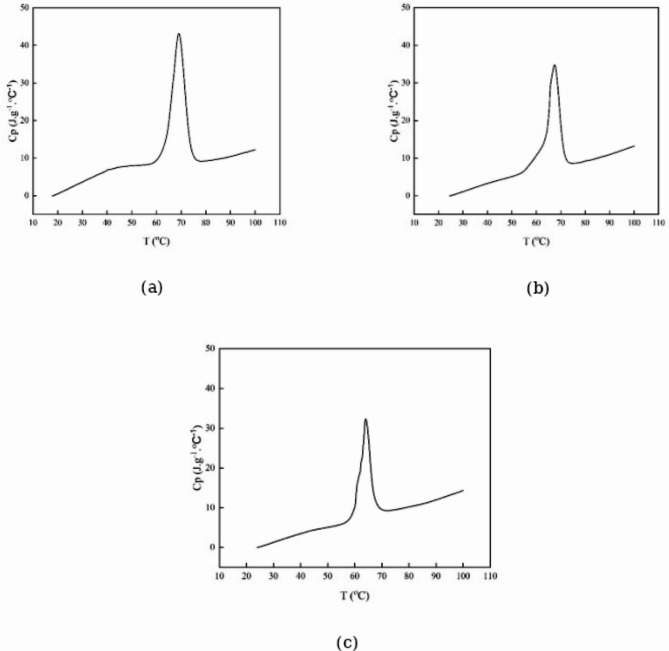
The thermograms of of the; (**a**) PCM1 ([HEA]La/SA), (**b**) PCM2 ([BHEA]La/SA), (**c**) PCM3 ([THEA]La/SA).

The specific heat capacity (*C*p) values of the PCMs were measured over a temperature range of 323.15 K to 353.15 K. The values, as presented in Table 3 and illustrated in Fig. 5, were obtained using DSC. The DSC is a thermal analysis technique that measures heat flow into or out of a sample as it undergoes temperature changes, allowing for the determination of heat capacity.

**Table 3:** The specific heat capacities (*Cp*) (J·g^-1^·ºC^-1^) of PCM1 ([HEA]La/SA), PCM2 ([BHEA]La/SA) and PCM3 ([THEA]La/SA) at the melting points and at different temperatures.

*T*/K	PCM1 ([HEA]La/SA)	PCM2 ([BHEA]La/SA)	PCM3 ([THEA]La/SA)
323.15	7.946	5.092	5.048
328.15	8.165	6.596	5.841
333.15	9.407	10.700	10.148
337.13	–	–	32.314
338.15	19.558	20.803	27.263
340.64	–	34.767	–
342.21	43.158	–	–
343.15	39.757	18.236	9.532
348.15	10.593	8.621	9.504
353.15	9.235	9.213	10.191

Even though all the studied PCMs contained the same amount of stearic acid as a base component, their specific heat capacities differed due to the influence of different ionic liquids (ILs) incorporated into the formulations. The type and properties of the ILs—such as molecular structure, polarity, and interaction with stearic acid—affect the material’s ability to store and transfer heat. This variation in heat capacity is critical because it determines how efficiently the PCM can absorb and release thermal energy.

Among the three studied PCMs, PCM1 ([HEA]La/SA) exhibited the larger *C*p values compared to the other two formulations. This suggests that the [HEA]La ionic liquid played a significant role in enhancing the heat storage capacity of PCM1, possibly due to stronger molecular interactions and high thermal conductivity between the IL and stearic acid. Such an increase in heat capacity could make PCM1 more efficient for thermal energy storage applications, where *C*p values contribute to improved thermal regulation and stability.

**Fig. 6:**
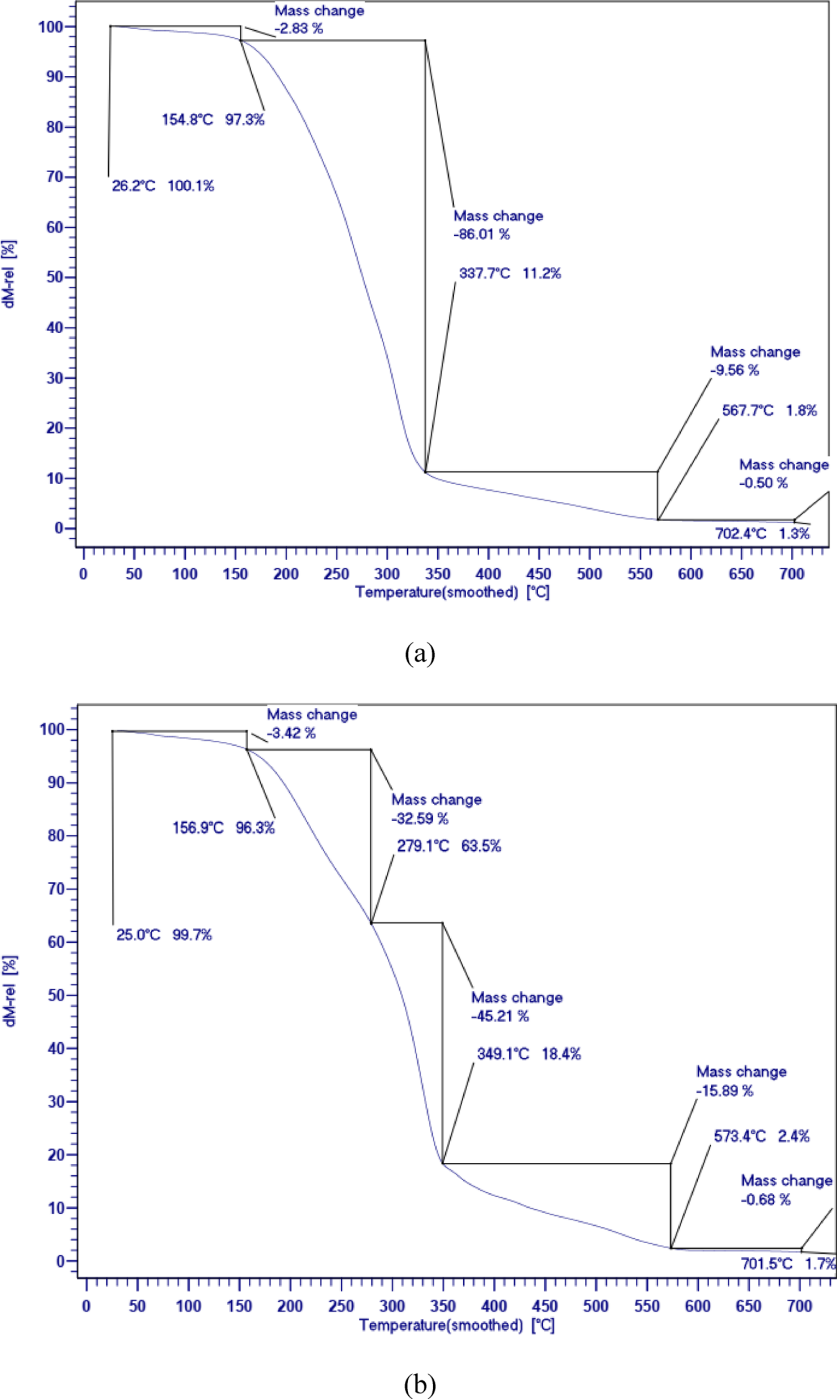

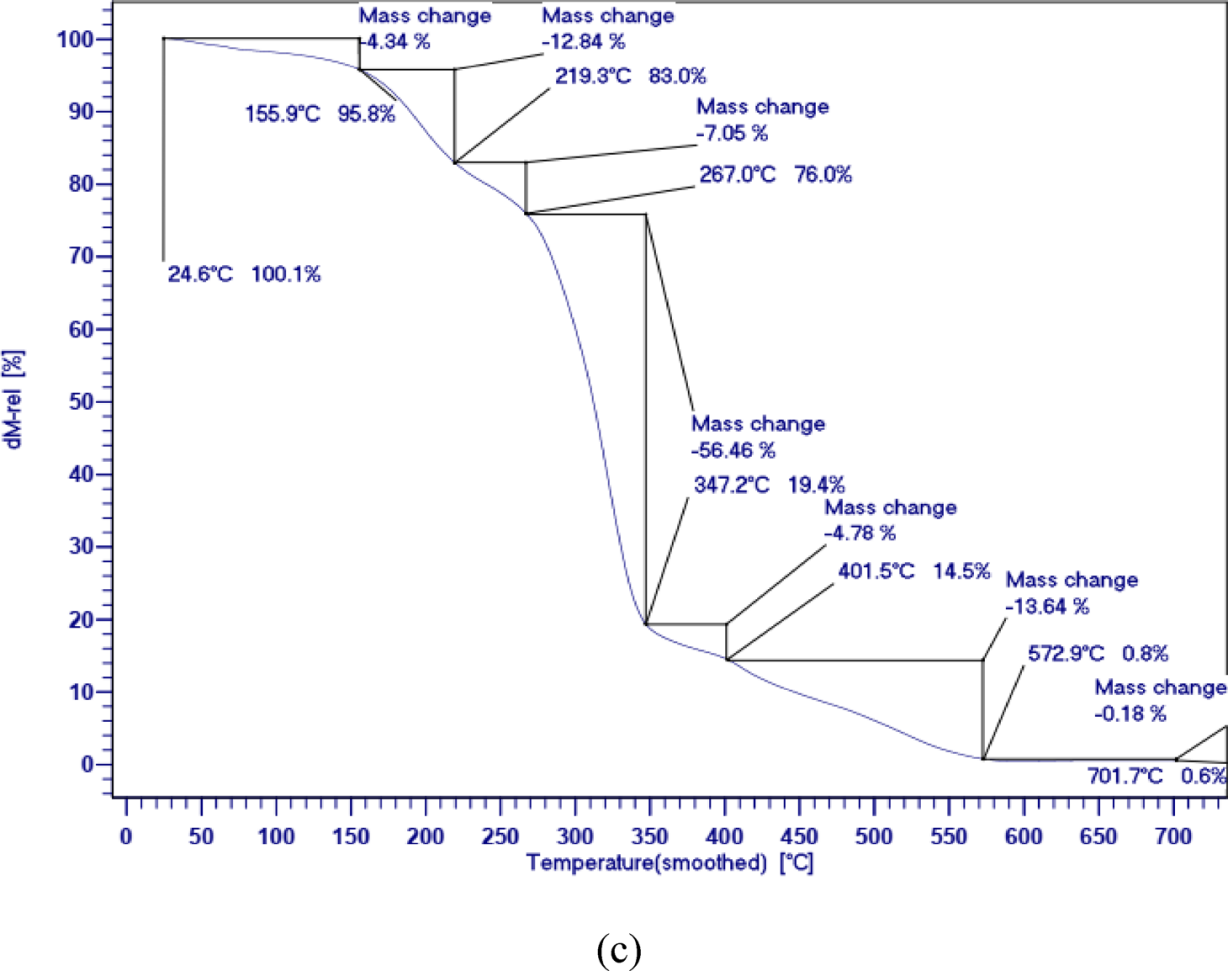
The quasistatic TGA of the; (**a**) PCM1 ([HEA]La/SA), (**b**) PCM2 ([BHEA]La/SA), (**c**) PCM3 ([THEA]La/SA).

Table 2 shows the residual value in the range of 50 to 700 °C and the weight loss temperature. Figure 6 displays the TGA curves for PCMs which shows the three-stage mechanisms of thermal degradation. During the three-step thermal degradation processes, the weight loss of PCM1 ([HEA]La/SA) is relatively lower than that of PCM2 ([BHEA]La/SA) and PCM3 ([THEA]La/SA). The first step, which is related to the release of water molecules absorbed in the network of ILs, is carried out between 293 and 373 K, as shown in Fig. 6. The second stage is related to the thermal destruction of molecular chains of ILs. Given that solar cell systems and solar thermal storage may operate at temperatures of up to 423.15 K, the synthesized PCMs show about 97% thermal stability, which is considered a suitable PCM. In comparison to the other two created PCMs, PCM1 ([HEA]La/SA) has the maximum thermal stability up to a temperature range of 423.15 K. The findings shown in Fig. 6 show that PCM1 ([HEA]La/SA), PCM2 ([BHEA]La/SA), and PCM3 ([THEA]La/SA) have, respectively, thermal stability values of 97.17, 96.58, and 95.66%.

The research study’s result is the implementation of ILs with favorable properties to improve PCMs made of fatty acids for thermal energy storage applications. As evidenced by FT-IR measurements, the combination of ILs with fatty acids did not induce any chemical reactions. Furthermore, the XRD and SEM data confirm that the resulting PCMs exhibit a layered and uniform microcrystalline structure. This layered structure, with a higher number of layers, contributes to the substantial energy release during the solid–liquid transition when subjected to heating.

The utilization of innovative materials characterized by substantial latent heats of fusion, elevated heat capacities, and remarkable thermal stability holds great promise for advancing thermal energy storage applications. Within this context, the PCMs developed in this study are introduced as novel materials distinguished by their high heat capacities and remarkable thermal stability, reaching up to an impressive 97.17%. These PCMs also exhibit a significantly greater latent heat of fusion, reaching approximately 245.00 kJ kg^-1^ when compared to the raw materials.

The key takeaway from this study lies in the effectiveness of these high-performance PCMs for thermal energy storage in solar cell applications. With the ability to capture and store heat effectively, solar cells may operate at higher temperatures, up to 423.15 K. The surplus heat, which would otherwise go unused, can be efficiently harnessed and converted into energy using PCMs. This approach holds the potential to eliminate the need for external power sources during nighttime hours, as the stored latent heat in the PCMs can be continuously released to meet energy demands, even in the absence of sunlight.

## Conclusions

This work presents a unique and environmentally friendly approach to thermal energy storage through the utilization of a latent heat process. In this study, the novel phase change composites (PCMs) were prepared using 2-hydroxyethylammonium-based ionic liquids (ILs) with a fatty acid, stearic acid. The incorporation of ILs markedly improved the structure, DSC results, and stability of the PCMs, enhancing their suitability for TES applications.

The melting point of the PCMs, specifically PCM1 ([HEA]La/SA), PCM2 ([BHEA]La/SA), and PCM3 ([THEA]La/SA), was determined to fall within the range of 337 to 342 K. Notably, PCM1 ([HEA]La/SA) exhibited the highest thermal stability, extending up to 423.15 K. This enhanced thermal stability and characteristics of PCM1 ([HEA]La/SA) could be attributed to the formation of hydrogen bonds between ILs and stearic acid. Additionally, the structure of PCM1 ([HEA]La/SA) appeared more compact and self-looped, requiring more energy to disassemble its structure. This structural feature, along with the presence of fewer hydroxyethyl branches leading to stronger hydrogen bonding, resulted in higher *C*p values for PCM1 ([HEA]La/SA) compared to the other two PCMs.

## Data Availability

All data generated or analyzed during this study are included in the published article.

## Abbreviations

PCM: Phase change materials
TES: Thermal energy storage
Ils: Ionic liquids
[THEA]La: Tris(2-hydroxyethylammonium) lactate
[BHEA]La: Bis(2-hydroxyethylammonium) lactate
[HEA]La: 2-Hydroxyethylammonium lactate
SA: Stearic acid
PCM1: [HEA]La/Sa
PCM2: [BHEA]La/Sa
PCM3: [THEA]La/Sa
XRD: X-ray diffraction
SEM: Scanning electron microscopy
FT-IR: Fourier transformation infrared spectroscopy
DSC: Differential scanning calorimeter
TGA: Thermogravimetric analysis
